# Intraoperative Capnothorax during Robotic Diaphragmatic Endometriosis Excision

**DOI:** 10.1155/2022/5935312

**Published:** 2022-04-26

**Authors:** Tyler Dunn, Lopa Misra

**Affiliations:** Mayo Clinic, Department of Anesthesiology and Perioperative Medicine, 5777 E, Mayo Blvd, Phoenix, AZ 85054, USA

## Abstract

Laparoscopic surgery is performed quite commonly and is known to have numerous advantages over traditional open surgery. Albeit rare, there are life-threatening complications as a result of laparoscopic surgery including those associated with the cardiopulmonary system. In our case, we present hemodynamically unstable capnothorax that occurred during robotic diaphragmatic endometriosis excision which was treated conservatively. It is critical for anesthesia providers to recognize when capnothorax occurs and to understand the implications and treatment in order to minimize unnecessary procedures and morbidity associated with such.

## 1. Introduction

The following case report will discuss the intraoperative and postoperative clinical course and management of capnothorax.

## 2. Case Presentation

**Figure 1:**
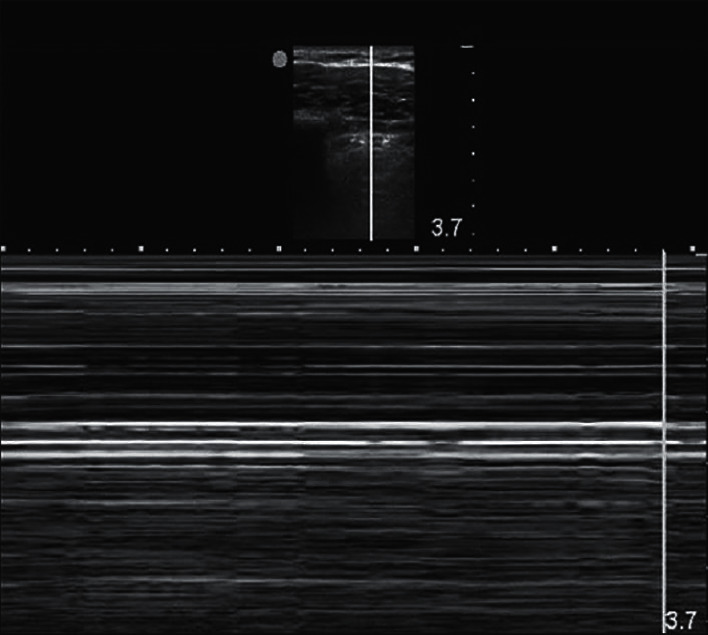
Transthoracic ultrasound showing “barcode” in M-mode from [Abdalla W., Elgendy M., Abdelaziz A. A., Ammar M. A. Lung ultrasound versus chest radiography for the diagnosis of pneumothorax in critically ill patients: a prospective, single-blind study. Saudi Journal of Anaesthesia 2016; 10 (3): 265–269].

A 34-year-old female with a medical history of chronic pelvic pain underwent endometriosis excision of pelvic, small bowel, and hemidiaphragmatic implants. Patient underwent standard induction with lidocaine, propofol, fentanyl, and rocuronium, and surgery began in the pelvis and progressed caudally. Approximately 3.5 hours into the case, the patient was placed in reverse Trendelenburg position and the surgeon focused attention on the diaphragmatic endometriosis implants. The right hemidiaphragm peritoneum was stripped from the musculature at the posterior aspect and excised. End tidal CO_2_ increased to the 60s followed by acute desaturation to the 80s and hypotension with systolic blood pressures in the 40s followed by end tidal CO_2_ dropping to the 20s. She was noted to have diminished right-sided breath sounds, and ultrasound demonstrated a “barcode” sign as shown in Figure 1. The robot was emergently undocked and insufflation was stopped. Patient was placed in Trendelenburg position, resuscitated with vasopressors and 5% albumin, and an arterial line was placed. The patient's hemodynamic and ventilatory parameters greatly improved, and the abdomen was closed. The patient was extubated, placed on supplemental oxygen via face mask, and chest X-ray was obtained showing a 3 cm pneumothorax in the right lung apex. A pigtail chest tube was planned to be placed by IR, but on chest CT prior to pigtail placement, the pneumothorax had considerably decreased. Given the rapid resolution, we suspected capnothorax was the ultimate cause of patient decompensation.

## 3. Discussion

### 3.1. Endometriosis

There are various locations in which endometriosis can be found within the body, all having their own specific anesthetic considerations. These anatomic locations include ovaries, ligaments of the uterus, fallopian tubes, perineum, intestinal tract, abdominal wall, liver, kidney, diaphragm, and lung tissue [1]. Surgery is performed either laparoscopically or robotically to excise or ablate the endometrial implants. If an endometrial implant is in lung tissue, then a video-assisted thoracoscopic surgery is typically performed, which may require a double lumen endotracheal tube.

### 3.2. Causes of Capnothorax

The incidence of pneumothorax ranges from 0.01 to 0.4% based on surgical procedure and patient comorbidities [2]. A capnothorax is a type of pneumothorax specifically from CO_2_ used for insufflation during laparoscopic procedures. These may be caused by a variety of factors including gas tracked through fascial planes from the neck and thorax into mediastinum and pleural space, dissection around diaphragm or from the passage of gas through the pleuroperitoneal hiatus, and congenital diaphragmatic defects. Congenital large diaphragmatic defects are rare, occurring in 2.3 out of every 10,000 births, while the occurrence of small asymptomatic defects is not known [3].

### 3.3. Presentation and Diagnosis of Capnothorax

The initial presentation of capnothorax can be nonspecific, but there are a few key features that narrow the presentation. The first sign may be increasing end tidal CO_2_ not responsive to ventilatory adjustments. This is followed by an increase in airway pressure and hypoxemia. On physical exam, subcutaneous emphysema of the head and neck may be present with unequal chest expansion and unilateral breath sounds [4]. If the surgeon can evaluate the diaphragm endoscopically, a bulging diaphragm can be seen [5].

A rapid confirmation of the diagnosis can be done intraoperatively with transthoracic ultrasound looking for lung sliding and M (motion) mode as well as a chest X-ray. Transthoracic ultrasound is best performed at the anterior chest wall lateral to the sternum in the second intercostal space, while the patient is supine, as air rises to the least dependent portion of hemithorax. Transthoracic ultrasound has the advantage of not only being a more portable and cost-effective tool for diagnosis, but it is also shown to be more accurate compared to X-ray with an 81% sensitivity and 100% specificity [6].

### 3.4. Treatment of Capnothorax

The treatment of capnothorax varies based on patient stability; with sound clinical evaluation of the patient, the provider may avoid unnecessary invasive treatments possibly resulting in increased morbidity and pain. If capnothorax has been identified in a hemodynamically stable patient, simple reduction of insufflation pressures, hyperventilation, increasing FiO_2_, and discontinuing nitrous oxide if used may be sufficient treatment. In a patient that begins to show hemodynamical effects of capnothorax, cessation of the CO_2_ pneumoperitoneum is critical in order to allow for spontaneous reabsorption of residual CO_2_ [7]. In addition, increasing PEEP on the ventilator will reduce the pressure gradient between the abdominal cavity and pleura which reduces the passage of gas to the pleura and helps inflate the lung [5, 8]. In a hemodynamically unstable patient that is unresponsive to conservative treatments, thoracentesis followed by chest tube placement for decompression is critical.

Given the high solubility of CO_2_, approximately 20 times that of nitrogen, it is relatively easy for the gas to readily diffuse across membranes. This leads to rapid resolution of capnothorax compared to that of pneumothorax. While the exact timing has not been studied in the literature, clinical evidence suggests that complete reabsorption and reexpansion of a compressed lung can occur within 30–60 minutes [9].

### 3.5. Summary

Overall, capnothorax is a relatively rare complication but can be life-threatening and have significant patient morbidity. Early recognition by the anesthesia provider coupled with effective and urgent communication with the surgical team is critical for timely intervention. Conservative treatments outlined above can spare patients more invasive and painful treatments such as chest tube placement. This highlights why CO_2_ is the gas of choice for maintaining pneumoperitoneum, specifically its high solubility.

## Data Availability

The deidentified patient data used to support the findings of this study are included within the article.
